# Unprecedented outbreak of respiratory syncytial virus in Taiwan associated with ON1 variant emergence between 2010 and 2020

**DOI:** 10.1080/22221751.2022.2054365

**Published:** 2022-03-31

**Authors:** Wei-Hsuan Lin, Fang-Tzy Wu, Yi-Yin Chen, Chih-Wei Wang, Ho-Chen Lin, Ching-Chia Kuo, Wan-Chun Lai, Fang-Ju Lin, Wan-Tin Tiew, An-Li Tsai, Kuan-Ta Ho, Ting-Yu Kuo, Chung-Hao Li, Ching-Yi Wu, Yi-Jiun Pan, Kuo-Chien Tsao, Yu-Chia Hsieh

**Affiliations:** a Department of Pediatrics, Chang Gung Children's Hospital, Chang Gung Memorial Hospital, College of Medicine, Chang Gung University, Taoyuan, Taiwan; b Center for Diagnostics and Vaccine Development, Centres for Disease Control, Ministry of Health and Welfare, Taipei, Taiwan; c Departments of Anatomic Pathology, Chang Gung Memorial Hospital, College of Medicine, Chang Gung University, Taoyuan, Taiwan; d Department of Pediatrics, Chang Gung Children's Hospital, Chang Gung Memorial Hospital, College of Medicine, Chang Gung University, Kaohsiung, Taiwan; e Department of Microbiology and Immunology, School of Medicine, College of Medicine, China Medical University, Taichung, Taiwan; f Research Centre for Emerging Viral Infections, Chang Gung University, Taoyuan, Taiwan; g Department of Laboratory Medicine, Linkou Chang Gung Memorial Hospital, Taoyuan, Taiwan

**Keywords:** Respiratory syncytial virus, genotypes, variant, G-protein, ON1

## Abstract

An outbreak of respiratory syncytial virus (RSV) has been observed in Taiwan since August 2020. We reviewed a central laboratory-based surveillance network established over 20 years by Taiwan Centres for Disease Control for respiratory viral pathogens between 2010 and 2020.

A retrospective study of children <5 years old hospitalized with RSV infection at Chang Gung Memorial Hospital between 2018 and 2020 was conducted, and samples positive for RSV-A were sequenced. Clinical data were obtained and stratified by genotype and year.

Data from 2020 showed an approximately 4-fold surge in RSV cases compared to 2010 in Taiwan, surpassing previous years during which ON1 was prevalent. Phylogenetic analysis of G protein showed that novel ON1 variants were clustered separately from those of 2018 and 2019 seasons and ON1 reference strains. The variant G protein carried six amino acid changes that emerged gradually in 2019; high consistency was observed in 2020. A unique substitution, E257K, was observed in 2020 exclusively. The F protein of the variant carried T12I and H514N substitutions, which weren’t at antigenic sites. In terms of multivariate analysis, age (OR: 0.97; 95% CI: 0.94–0.99; *p*  = 0.02) and 2020 ON1 variant (OR:2.52; 95% CI:1.13–5.63; *p* = 0.025) were independently associated with oxygen saturation <94% during hospitalization.

The 2020 ON1 variant didn’t show higher replication or virulence compared with those in 2018 in our study. The unprecedented 2020 RSV epidemic may attribute to antigenic changes and lack of interferon-stimulated immunity induced by seasonal circulating virus under non-pharmaceutical intervention.

## Introduction

In view of the international epidemic caused by coronavirus disease (COVID-19) in 2020, Taiwanese officials implemented strict prevention measures (including mask wearing, handwashing and social distancing). Although schools have remained open, it significantly reduced the spread of influenza and enterovirus known to circulate annually [1]. In addition, strict quarantine for overseas arrivals has been conducted, with a persistent decrease in visitor numbers. However, an enormous surge in cases of respiratory syncytial virus (RSV) infection among children in Taiwan has been observed since August 2020.

RSV, a negative-strand, nonsegmented RNA pneumovirus of the family *Pneumoviridae* is the most common viral pathogen detected in young children with pneumonia and bronchiolitis [2,3]. Moreover, in children hospitalizations due to RSV are associated with chronic wheeze and asthma [4]. Globally, RSV resulted in approximately 33.1 million episodes of acute lower respiratory infection with 3.2 million hospital admissions and 59600 in-hospital deaths among children younger than 5 years throughout 2015 [5]. RSV contains 10 genes that are translated into 11 proteins, two of which are major antigenic surface proteins: the G attachment protein mediates virus binding, and the F fusion protein intervenes in subsequent membrane fusion [6]. In contrast to the highly conserved F protein, the evolution of RSV revealed preferential diversification of the G protein under the host’s immunological pressure. G proteins are classified as A and B types based on the genetic variability of the second hypervariable 2 region, which are further divided into 16 RSV-A genotypes and 23 RSV-B genotypes [7].

Usually, RSV-A or B predominates in a single season, and they alternate or co-circulate annually. Outbreaks of RSV are often the result of variants that develop from the locally evolved clades, replacing the previously circulating RSV genotype. For instance, a novel genotype ON1 of RSV-A, with a 72-nt duplication within the highly variable G gene, was first reported in Ontario, Canada in December 2010, which has rapidly spread and diversified into several variants worldwide [8]. The additional 72-nt duplication in ON1 of RSV-A viruses provides variable amino acid changes, subsequently associated with antigenic diversity, increased fitness, increased severity, and immune evasion over previous RSV-A genotypes [9–11].

In Taiwan, RSV-A and B co-circulated with alternative dominance between 2000 and 2016 [12]. ON1 of RSV-A first circulated in Taiwan in 2011. Since 2013, ON1 has almost completely displaced the previously circulating RSV-A NA1 with a prevalence rate of approximately 25–50% compared to RSV-B [12]. According to the surveillance data that monitored emergency and outpatient visits for patients with acute infections from the Taiwan Centres for Disease Control, RSV was never included as the top three respiratory pathogens between 2010 and 2019. At the end of 2020, the surveillance system demonstrated that RSV was the top respiratory pathogen causing a large epidemic that started in August, achieving a peak in November.

In this study, we report the annual activity of RSV from 2010 to 2020 based on surveillance data in Taiwan. The clinical and molecular characteristics of RSV strains associated with the 2020 epidemic were compared with those of previously circulating genotypes collected from two medical centres.

## Materials and methods

### Ethical approval

This study was approved by the Research and Ethics Committee of Chang Gung Memorial Hospital (IRB: 202100450B0, 202100569A3). The animal testing procedures used in this study were reviewed and approved by the Institutional Animal Care and Use Committee (IACUC) of Chang Gung Memorial Hospital (Approval Number: 2021032305), and the proposed animal experiment follows the guidelines outlined in the Guide for Laboratory Animal Facilities and Care as promulgated by the Council of Agriculture. Executive Yuan, ROC. All animals were housed in an animal facility at 22 °C, with a relative humidity of 55%, in a 12 h light/12 h dark cycle, with sterile tap water and food available ad libitum.

### Surveillance network and viral isolation and identification

A laboratory-based surveillance network monitoring influenza was established in Taiwan in 2000 [1]. The community surveillance network is coordinated by the Taiwan Centres for Disease Control and collaborated with 8–11 virology laboratories with approximately 750 sentinel sites distributed in northern, central, southern, and eastern Taiwan. Routinely, throat swabs or nasopharyngeal aspirates were obtained from hospitalized patients and outpatients with influenza-like infection symptoms. Infected patients with one or more of these symptoms were included in this study. Please refer to Supplemental Appendix for more detailed methods for viral isolation from specimens.

### Study design and population

We conducted a retrospective study of children hospitalized with RSV infections from 2018 to 2020 at Chang Gung Memorial Hospital (CGMH)-Linkou branch, a 3,700-bed medical centre in northern Taiwan, and CGMH-Kaohsiung branch, a 2700-bed medical centre in southern Taiwan. Children <5 years of age with culture-confirmed RSV infections were enrolled. Patients with underlying diseases such as prematurity, chronic lung disease, congenital heart disease, genetic metabolic disorder, nervous system disease, immune deficiency, cancer, or incomplete medical records were excluded. Patients were classified into 3 groups: ON1 infection in 2018 and 2019 (February 2018–January 2020), ON1 infection in 2020 (February 2020–January 2021), and RSV-B infection between 2018 and 2020 (February 2018–January 2021).

### RSV typing and sequencing

All RSV-A strains were sequenced in part of the G conserved region and the second hypervariable region up to the stop codon, and 29 strains of RSV-A were randomly selected for the full-length F gene sequencing. These sequences were deposited in GenBank database (accession nos. MZ417560–MZ417781; Tables S1 and S2). Sequences were aligned with reference ON1 strains from Taiwan between 2011 and 2016 and ON1-1.1–ON1-1.4 worldwide using MEGA 6, which were constructed in phylogenetic tree. More details are provided in Supplemental Appendix.

### Glycosylation site prediction

The bioinformatics tools NetNGlyc and NetOGlyc (http://www.cbs.dtu.dk/services) were used to predict N- and O-linked glycosylation sites in the ON1 amino acid sequences.

### Animals and cells

BALB/cByJNarl mice were provided by the animal centre of NARLabs (NLAC, Taiwan). HEp2 and MRC5 cells were maintained in minimum essential medium (MEM, Gibco). All cell-cultured medium were supplemented with 10% fetal bovine serum (FBS, Gibco) and 1% penicillin/streptomycin (P/S, Gibco) at 37°C in a humidified incubator at 5% CO_2_ and subcultured twice a week.

### Viral infection on cells

Approximately 2×10^5^ cells were seeded onto each well of 12-well plates and cultured at 37°C, 5% CO_2_ for 16 h. Two RSV-A viruses including one in 2018 (RSV-A 51031) and the other in 2020 (RSV-A E115) were inoculated into the cells at 2×10^7^ RNA copies. The virus was incubated with the cells at 37 °C, 5% CO_2_ for 2 h, after that 0.9 mL medium (MEM with 2% FBS and 1% P/S) was added into each well. At 0, 48, 72, and 168 h after infection, the viral RNA of culture supernatants were collected and determined using RT-qPCR.

### Fitness in a mouse model

Eight- to ten-week-old female BALB/c mice were inoculated intranasally with a 50 μL volume of 1 × 10^7^ RNA copies of RSV-A; mock mice were inoculated with MEM only. Infected mice were weighed and monitored daily for signs of illness. On days 2, 4, and 6 after infection, mice were euthanized via decapitation after administering anaesthesia using isoflurane, according to the guidelines issued by the Institutional Animal Care and Use Committee (IACUC) of Chang Gung Memorial Hospital, and the nasal washes were collected in 0.5 mL MEM. The whole lung was collected and homogenized in MEM and stored at −80 °C until the RT-qPCR analysis. Viral RNA was directly extracted from mouse lung homogenates and nasal washes.

### Statistical analyses

Statistical significance was set at *p* < 0.05. All statistical analyses were performed using IBM SPSS Statistics for Windows, version 26.0.

## Results

### Surveillance data

**Figure 1.**
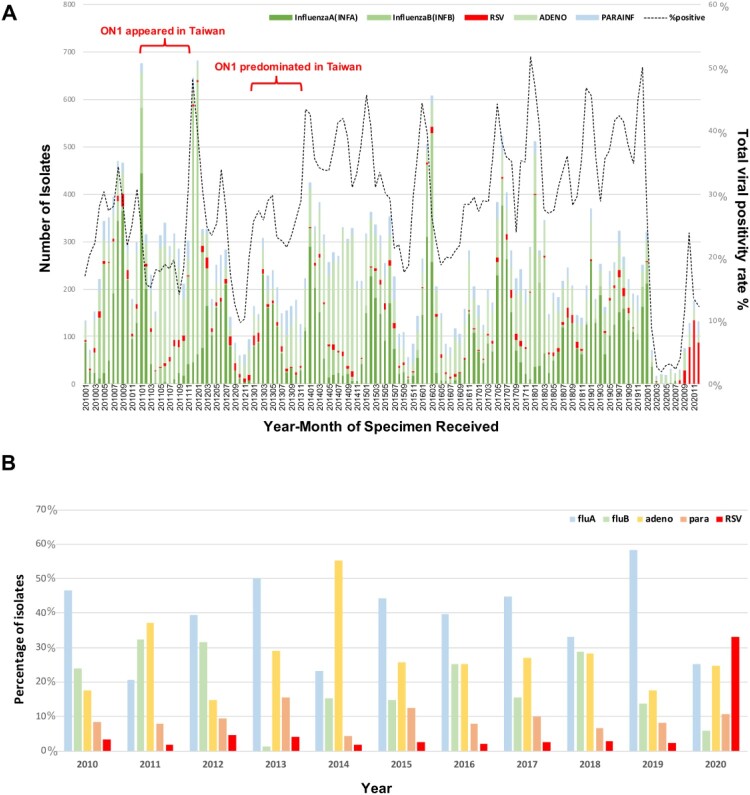
(A) Monthly respiratory virus isolation between 2010 and 2020 from central laboratory-based surveillance network established by Taiwan Centres for Disease Control (TCDC). The respiratory viral positivity rate has declined remarkably since February 2020. Influenza virus types A and B accounted for the largest proportion from 2010 to 2019. However, respiratory syncytial virus incidence exhibited an enormous increase since August 2020, even with a lower viral positivity rate. X-axis: year-month of specimen received, left Y-axis: number of isolates, right Y-axis: total viral positivity rate, dashed black line: the viral positivity rate between 2010 and 2020. (B) The top five virus isolated from respiratory tract between 2010 and 2020 (b). The most common respiratory viruses in the past ten years included influenza A and B and adenoviruses. In 2020, respiratory syncytial virus became the most common respiratory virus, replacing influenza virus and adenovirus. Data obtained from the Taiwan Centres of Disease Control. Y-axis: percentage of isolates, X axis: year.

Approximately 22 to 44% of nasopharyngeal specimens collected for viral culture were positive for at least one respiratory virus in the past 10 years since 2010; this number decreased to 13.5% in 2020 (Figure 1A and Table 1). Influenza virus A or B accounted for the largest proportion of positive samples obtained from 2010 to 2019, excluding 2011 and 2014, during which adenovirus accounted for the largest proportion, followed by influenza virus. However, RSV infection has increased significantly since August 2020, becoming the most common respiratory viral pathogen in 2020 (Figure 1A,B). Among these samples positive for viral pathogens in the past 10 years, RSV accounted for <5% between 2010 and 2019 but reached 33.2% in 2020 (Figure 1B). We analyzed the annual positivity rate of RSV, adenovirus, and influenza virus in the past 10 years and compared each year with 2010, which was set as the reference year. In 2011, when ON1 initially appeared in Taiwan, the RSV positivity rate had a decrease compared with 2010 (OR 0.433, 95% CI 0.32–0.58, *p* <0.001), and in 2012, it was 1.4- fold greater than that in 2010 (OR 1.422, 95% CI 1.1–1.83, *p* = 0.006). After, no obvious RSV epidemic occurred in Taiwan, and the number of RSV infections reached the minimum in 2016 (OR 0.686, 95% CI 0.49–0.95, *p* = 0.021). However, in 2020, the RSV positivity rate surged (Figure 1A) and reached 3.7-fold higher than in 2010 (OR 3.79; 95% CI, 3.06–4.69; *p* <0.001) (Table 1).

**Table 1.** The surveillance data for respiratory virus isolation from 2010 to 2020 and annual odds ratio of RSV, adenovirus, influenza A, B cases compared to the reference year 2010.

Year	No. of total samples/Viral positive rate (%)	No. of positive results/rate (%)							Odds ratio (95% confidence interval)				
		RSV	Adenovirus	Influenza A	Influenza B	RSV	*P* value	Adenovirus	*P* value	Influenza A	*P* Value	Influenza B	*P* value
2010	12165 (31.7)	113 (0.92)	592 (4.86)	1572 (12.9)	808 (6.64)				Reference				
2011	19280 (22.4)	76 (0.39)	1513 (7.84)	839 (4.35)	1317 (6.8)	0.433 (0.32–0.58)	**<0**.001*	1.67 (1.51–1.83)	**<0**.001*	0.31 (0.28–0.34)	**<0**.001*	1.03 (0.94–1.13)	0.5
2012	9955 (31.9)	129 (1.29)	406 (4.07)	1091 (10.96)	873 (8.77)	1.422 (1.1–1.83)	**0**.01*	0.83 (0.73–0.95)	**0**.01*	0.83 (0.76–0.9)	**<0**.001*	1.35 (1.23–1.50)	**<0**.001*
2013	8524 (30.4)	93 (1.09)	660 (7.74)	1138 (13.35)	30 (0.35)	1.202 (0.91–1.58)	0.189	1.65 (1.47–1.85)	**<0**.001*	1.04 (0.96–1.13)	0.35	0.05 (0.04–0.08)	**<0**.001*
2014	9447 (41.9)	68 (0.71)	2018 (21.3)	843 (8.92)	556 (5.89)	0.835 (0.62–1.13)	0.24	5.65 (5.13–6.22)	**<0**.001*	0.69 (0.64–0.76)	**<0**.001*	0.93 (0.83–1.04)	0.19
2015	8398 (35.1)	70 (0.83)	685 (8.15)	1176 (14)	394 (4.69)	0.922 (0.69–1.24)	0.59	1.74 (1.55–1.95)	**<0**.001*	1.09 (1.01–1.19)	**0**.02*	0.69 (0.62–0.78)	**<0**.001*
2016	8764 (35.3)	54 (0.61)	692 (7.89)	1096 (12.5)	691 (7.88)	0.686 (0.49–0.95)	**0**.02*	1.68 (1.50–1.88)	**<0**.001*	0.96 (0.89–1.05)	0.4	1.21 (1.08–1.34)	**<0**.001*
2017	9063 (38.3)	86 (0.94)	861 (9.5)	1436 (15.84)	497 (5.48)	1.05 (0.79–1.38)	0.76	2.06 (1.85–2.29)	**<0**.001*	1.27 (1.18–1.37)	**<0**.001*	0.82 (0.73–0.92)	**0**.001*
2018	7568 (40)	83 (1.09)	794 (10.49)	934 (12.34)	812 (10.7)	1.21 (0.91–1.61)	0.18	2.29 (2.06–2.57)	**<0**.001*	0.95 (0.87–1.04)	0.25	1.69 (1.53–1.86)	**<0**.001*
2019	6942 (44.9)	66 (0.95)	508 (7.31)	1681 (24.21)	399 (5.74)	1.05 (0.78–1.43)	0.73	1.55 (1.38–1.75)	**<0**.001*	2.15 (1.99–2.32)	**<0**.001*	0.86 (0.76–0.97)	**0**.02*
2020	10028 (13.5)	342 (3.41)	256 (2.55)	259 (2.58)	62 (0.61)	3.79 (3.06–4.69)	**<0**.001*	0.52 (0.45–0.60)	**<0**.001*	0.18 (0.16–0.20)	**<0**.001*	0.09 (0.07–0.12)	**<0**.001*

*p < 0.05, No.: number, RSV: respiratory syncytial virus.

### Virologic surveillance for patients

**Figure 2.**
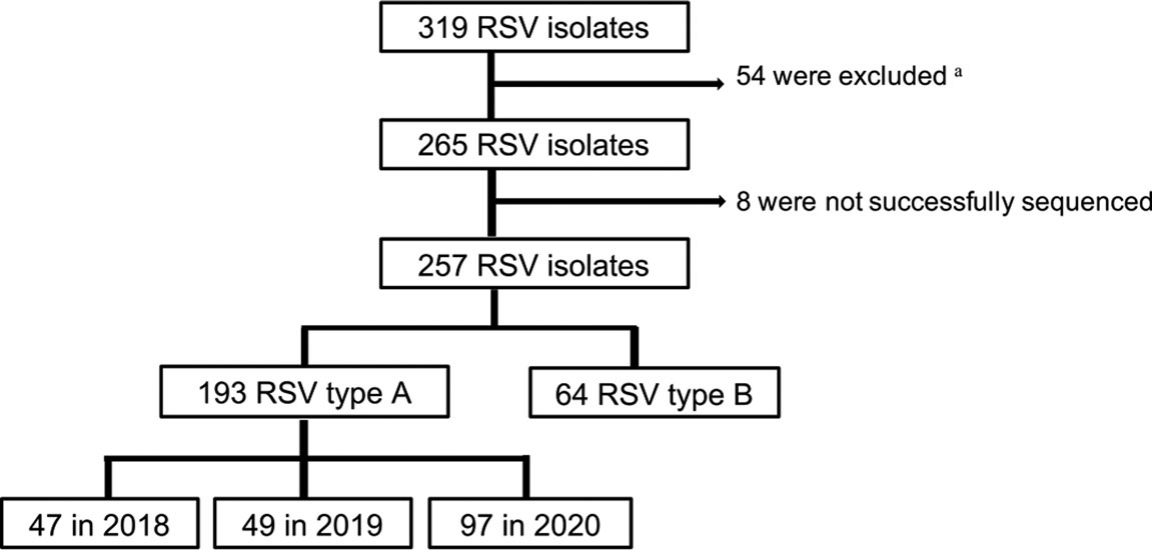
Flow chart describing cases selection. ^a^: patients without complete medical data, children younger than 5 years old, patient with underlying disease such as prematurity, chronic lung disease, congenital heart disease, genetic metabolic disorder, nervous system disease, immune deficiency or cancer were excluded.

Children <5 years of age hospitalized with respiratory symptoms between February 2018 and January 2021 were tested for respiratory viruses. After excluding patients with underlying disease and those without data available, 265 nasopharyngeal samples were found to be positive for RSV; of these, 257 strains were successfully sequenced and classified as 193 RSV-A and 64 RSV-B (Figure 2). In a total of 193 RSV-A strains, 47 strains were in 2018, 49 in 2019, and 97 in 2020, accounting for 58, 62.8, and 97.9% in each year, respectively. All RSV-A belonged to ON1 strains. Four major amino acid changes were noticed in G protein of ON1 strains in 2018, which included T200P, P215L, N255D, and V279I. Both substitutions T200P and N255D accounted for 34% in a total of 47 strains in 2018, 42% with P215L, and 38% with V279I. Of these amino acid changes, T200P and P215L were located in the heparin-binding domain, while N255D and V279I were in the second hypervariable sites of the G protein. In 2019, 6 amino acid substitutions occurred. Substitutions T113I, V131D, N178G, H258Q, and H266L were seen in 63% of the total 49 ON1 strains, and Y304H appeared in 30%. These substitutions appeared in 2018 ON1 strains but accounted for only 6%–23%. Moreover, in 2020, in addition to the 6 substitutions already noticed in 2019, the novel substitution E257K was first detected. In a total of 97 ON1 strains in 2020, substitutions T113I, V131D, N178G, H258Q, and H266L accumulated in all strains, while E257K and Y304H appeared in 90 and 98%, respectively. All denoted substitutions were located in the ectodomain. All but one substitution in 2019 and 2020 were found in mucin domains I (T113I, V131D) and II (E257K, H258Q, H266L, Y304H); while N178G was found in the central conserved domain.

With regards to F protein, substitutions A08T, F20L, S101P, N105S, K124N, R213S, N276S, V384I, H515N, and S540A appeared between 2018 and 2020, of which R213S and N276S were in antigenic sites. In addition, substitutions T12I and H514N in the F protein first emerged in 2020.

**Figure 3.**
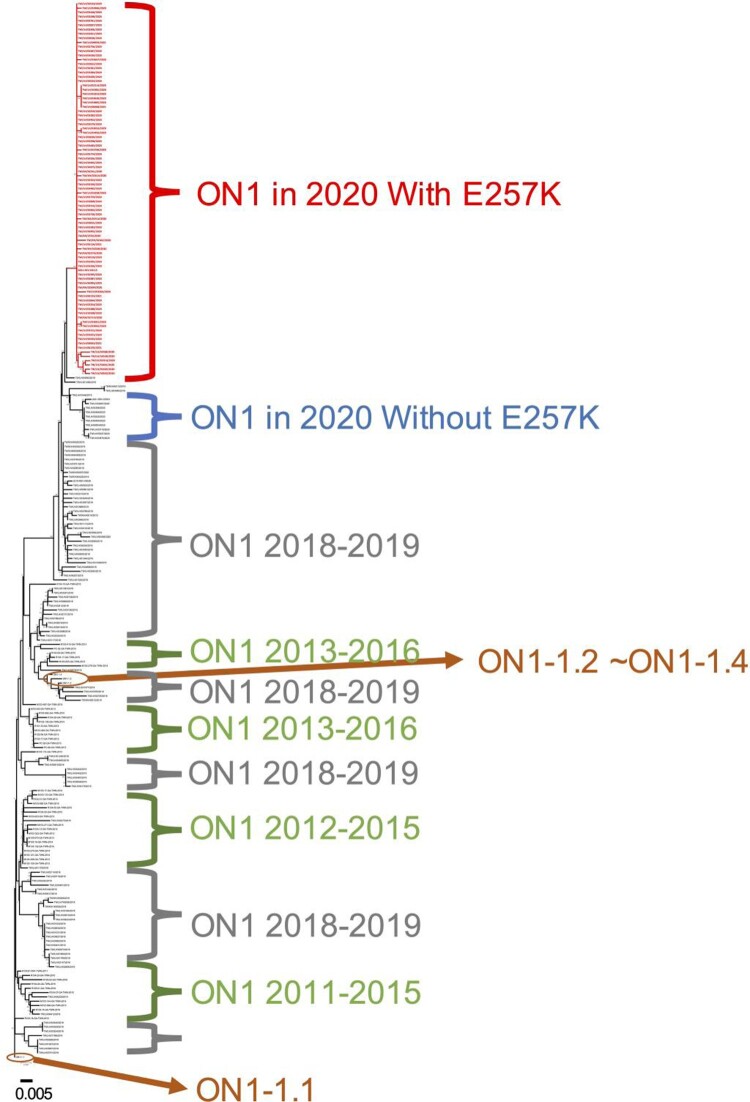
Phylogenetic analysis of the G gene of respiratory syncytial virus (RSV) ON1 strains circulating in Taiwan between February 2018 and January 2021 with reference strains between 2011 and 2016. The phylogenetic tree of G protein shows that ON1 samples from 2020 with amino acid substitution E257 K were clustered apart from those of the previous seasons including ON1 in 2020 without E257 K, ON1 in 2018 and 2019 in our study and reference strains between 2011 and 2016 in Taiwan. Our strains from 2020 were also apart from the reference strains ON1-1.1–ON1-1.4 from 2018 and 2019 seasons distributed in all clades. GenBank accession numbers used in this figure are listed in Table S1. Scale bar shows the number of substitutions per site.

Phylogenetic reconstruction of G protein sequences from this study with other 46 ON1 reference strains in Taiwan between 2011 and 2016 and ON1-1.1–ON1-1.4 worldwide in Figure 3 shows that strains of the 2018 and 2019 seasons distributed in all clades and clustered apart from previous reference strains. Interestingly, the ON1 specimens from the 2020 season formed a unique cluster that branched off the 2018 and 2019 seasons and other reference strains. Moreover, our strains in 2020 were also different from the reference strains ON1-1.1–ON1-1.4, which have been previously reported worldwide, indicating that a novel ON1 variant has been circulating in the community associated with the 2020 RSV epidemic in Taiwan (Figure 2). In addition, the phylogenetic tree of randomly selected 29 F protein sequences each year and reference strain JX198112.1 also showed that the strain in 2020 exhibited different clusters from those observed in 2018 and 2019 (Figure S1 and Table S2).

Amino acid sequences were submitted to online bioinformatics tools (NetNGlyc and NetOGlyc) to predict *N*- and *O*-linked glycosylation sites. Four amino acid sites were predicted to be N-glycosylated within the G protein of the ON1 strain. Of these, 103, 135 and 237 amino acid sites were predicted to be N-glycosylated in 2018, 2019, and 2020, respectively. In addition, RSV-ON1 strains in 2020 acquired one more N-glycosylation at amino acid sites 179, which did not appear before 2020. Furthermore, strains belonging to ON1 in 2020 in our study were characterized by the acquisition of 82–86 NetOGlyc sites similar to ON1 in 2018 and 2019, which was also similar to our reference strain JN257693.1.

We calculated the average p distance in each year from 2018 to 2020 and between each year and the following one (Table S3). Both analyses suggested an increasing genetic divergence of the ON1 strains during the study period.

### Clinical cases surveillance for inpatients

We analyzed the demographic data of 257 RSV-infected children younger than 5 years of age with respiratory tract infections between ON1 infection in 2018 and 2019, ON1 in 2020 and RSV-B from 2018 to 2020 (Table 2). The three groups comprised 96, 97, and 64 patients, respectively. No differences were found in terms of sex, age, duration of fever, wheezing, presence of chest retraction, receiving antibiotics, and PICU admission between the three groups. In 2020, most patients were infected with RSV-ON1; these patients presented O_2_ saturation <94% (*p* = 0.049) and complications (*p* = 0.014), including acute otitis media (AOM), pneumonia patch, hepatitis, and myocarditis. Moreover, AOM was more prevalent in ON1 patients infected in 2020 (*p* = 0.04), and the minimum O_2_ saturation during hospitalization was lowest in ON1 infected patients in 2020, with borderline statistical significance (*p* = 0.058). White blood cell count was the highest in RSV-B-infected patients (*p* = 0.02).

**Table 2.** Analysis of patients’ demographic and clinical Data in RSV positive patients grouped by RSV genotype and years.

Demographics	*N* (%)			
	RSV-ON1 2018–2019	RSV-ON-1 2020	RSV-B 2018–2020	*P* value
Case number	96	97	64	
Male (%)	56 (58.3%)	60 (61.8%)	38 (59.4%)	0.878
Age, median month (interquartile range)	19.95 ± 17.69	19.78 ± 14.28	16.34 ± 13.6	0.29
Fever Duration (mean ± SD (days))	3.45 ± 2.62	3.27 ± 2.42	3.16 ± 2.53	0.76
Fever >5 days	34 (35.4%)	32 (32.9%)	21 (32.8%)	0.92
Wheezing	34 (38.3%)	44 (44.4%)	22 (34.4%)	0.255
Retraction	16 (16.7%)	19 (19.1%)	11 (17.2%)	0.858
O_2_ saturation <94%	11 (11.4%)	23 (23.7%)	8 (12.5%)	**0**.049*
O_2_ saturation in minimum	95.4 ± 2.09	94.8 ± 1.59	95.5 ± 2.04	0.058
Complication	2 (2.1%)	11 (11.3%)	2 (3.1%)	0.014*
Acute otitis media	1 (1%)	5 (5.1%)	1 (1.5%)	0.04*
Pneumonia patch	1 (1%)	4 (4.1%)	1 (1.5%)	0.41
Hepatitis	0 (0%)	1 (1%)	0 (0%)	0.437
Myocarditis	0 (0%)	1 (1%)	0 (0%)	0.437
PICU admission	4 (4.2%)	4 (4.1%)	2 (3.1%)	0.935
Antibiotics treatment	57 (59.4%)	58 (59.8%)	36 (56.3%)	0.864
Mortality	0 (0%)	0 (0%)	0 (0%)	–
Lab				
CRP (mg/L) mean	19.66 ± 32.42	11.7 ± 21.69	20.15 ± 34.63	0.105
WBC/mm^3^ mean	9917 ± 4118	8846 ± 3712	10639 ± 4467	0.02*

*p <0.05; N: numbers; SD: standard deviation; CRP: C-reactive protein; WBC: white blood cell; PICU: pediatric intensive care unit; –: no available data.

The logistic regression analysis for factors associated with O_2_ saturation <94% is shown in Table 3. In the univariate analysis, age, wheezing, and ON1 variant in 2020 were significant risk factors. In the multivariate analysis, age (OR 0.97, 95% CI 0.94–0.99; *p* = 0.02) and ON1 variant in 2020 (OR 2.52; 95% CI 1.13–5.63; *p* = 0.025) were independently associated with O_2_ saturation <94% during hospitalization.

**Table 3.** The univariate and multivariate analysis by logistic regression for the factors associated with O_2_ saturation <94%.

Risk factor	Univariate analysis		Multivariate analysis	
	Odds ratio (95% CI)	*P* value	Odds ratio (95% CI)	*P* value
Sex(male)	1.6 (0.79 − 3.26)	0.19	–	–
Age(years)	0.97 (0.95 − 0.96)	0.018	0.97 (0.94 − 0.99)	0.02
Wheezing	1.93 (0.99 − 3.76)	0.053	1.57 (0.79 − 3.12)	0.2
ON1 in 2020	2.4 (1.09 − 5.25)	0.028	2.52 (1.13 − 5.63)	0.025
WBC/mm^3^ mean	1 (1.0 − 1.0)	0.619	–	–
CRP (mg/L)	0.99 (0.96 − 1.00)	0.772	–	–

Variables with *p* < 0.1 in the univariate analysis were included in the multivariate analysis. CI: confidence interval; –: no data in multivariate analysis.

### In vitro and in vivo models for studying 2018/RSV-A and 2020/RSV-A

We performed experiments both in cultured cells and in a mouse model to test whether 2020/RSV-A has a fitness advantage over 2018/RSV-A. In contrast to our clinical results, 2018/RSV-A showed a higher replication kinetics than that of 2020/RSV-A at 72 and 168 h post-infection in HEp2 cell (Figure S2). However, in the BALB/c model, body weight changes, viral titers from nasal washes and the lungs, and histologic scoring of lung tissue for epithelial damage, interstitial cellularity, and peribronchovascular infiltrates between 2018/RSV-A and 2020/RSV-A were not significantly different on day 2, 4, and 6 after infection (*p* >0.05) (Table S4 and Figures S3–S5).

## Discussion

This study evaluated the largest outbreak caused by a novel RSV-A ON1 variant during the past ten years in Taiwan. The magnitude and scale of the 2020 novel variant was approximately 4-fold greater than those in 2010 and 2013, when the original ON1 was the last predominant. These findings suggest that ON1 in Taiwan might have evolved into a new subgenotype, allowing it to supplant previously existing viral strains.

The RSV genome is error-prone due to the lack of proofreading mechanism of RNA-dependent RNA polymerase, allowing for rapid generation of single nucleotide polymorphisms and leading to altered virus pathogenesis and transmissibility [13]. The reemergence of rapidly evolving RSV poses a significant threat to global health. Whether disease severity differed between RSV-A ON1 and non-ON1 genotypes remains inconsistent. Yoshihara et al. reported that ON1 was associated with a higher risk of hospitalization and lower respiratory tract infection in children under 5 years old [11], whereas Panayiotou et al. and Midulla et al. demonstrated that infants or children under 2 years of infection with ON1 experienced milder acute respiratory infections than those infected with RSV NA1 [14,15]. Others found no significant difference in the clinical courses between ON1 and non-ON1 [16]. Along with the local spread of ON1 in each area, hundreds of ON1 divergent variants have been reported worldwide [10]. Of note, ON1 variants with specific amino acid changes were related to increased disease severity in Italy and the Netherlands [9,17]. The ON1.1 variant with a set of six amino acid substitutions (T200P, P215L, N255D, S275N, N279I, and E295V), ON1.2 variant with L274P and L298P changes, and ON1.3 variant with I243S and E262K changes were prevalent genotypes during the 2017/2018 season in Italy [9]. The ON1 variant circulating during the 2016/2017 season in the Netherlands had a set of eight novel amino acid substitutions (S102F, K216N, P256S, H258Y, S270Y, E271K, P300S, and Y304H) of the G protein [17]. ON1 evolving to ON1.4 with I/T136T and P206Q changes abruptly expanded in Kenya during 2014/2015, of which patients with ON1 infection had higher prevalence of intolerance to eat than those of non-ON1 infected patients [10].

These characteristic amino acid substitutions not only contribute to antigenic escape but also acquire more N- or O-glycosylation sites, which may trigger an innate antiviral immune response or promote viral survival and virulence [18]. The novel ON1 variant in this study carried one amino acid change in the central conserved domain and six amino acid changes in the bilateral highly glycosylated mucin domain, which makes the virus acquire one more N-glycosylation. We found that these amino acid changes not reported before with one more N-glycosylation were associated with disease severity. The unique combination of amino acid changes harboured by the novel RSV-A ON1 variant in this study suggests a potential structure or functional relationship of these amino acids, contributing to viral epidemics and independent association with O_2_ <94%.

Because of the relative genetic stability shown by F protein, vaccine and monoclonal antibody development for prophylaxis targets either the prefusion or postfusion form of the F protein, which contains six antigenic sites (I to V, Ø). To date, no RSV vaccine has been licensed for distribution. Prophylactic neutralizing monoclonal antibodies include palivizumab and motavizumab (site II; amino acid position 254–277), MK-1654 (site IV; amino acid position 422–471), nirsevimab (site Ø; amino acid positions 62–96, 195–227), and suptavumab (site V; amino acid positions 55–61, 146–194, 287–300). The latter two antigenic sites (Ø and V), which elicit the greatest frequency of high-potency antibodies, are present only in the prefusion form of the F protein [19]. Variability in the F protein could result in a virus that escapes neutralization by anti-RSV antibody [20]. A global molecular epidemiology assessment of the evolution of the F protein from 2017 to 2018 found that only 0.8 to 9.4% of RSV-A ON1 F exhibited amino acid changes (Y33H, T72A, N88T, I206T, S255N, S276N, S276R, I384 T, S425T, S466N, and L467I), which were detected in four antigenic sites [21]. In the United States, K68N and I206T at antigenic site Ø and S276N at antigenic site II were detected in 1–2.1% of RSV-A ON1 F from 2015 to 2017 [22]. Fortunately, despite a few sequence variations in the F gene, there was no amino acid substitution at the six antigenic sites in 2020 ON1 variant in this study.

The limitations of this study are the small sample size, retrospective study, and lack of whole-genome sequencing, which would have completed the picture of ON1 variability. Nevertheless, we have surveillance data to observe the trend of RSV infection. The Taiwan Centres for Disease Control has monitored inpatient and outpatient visits for patients with acute influenza-like infections for more than 10 years, maintaining a high level of awareness of emerging respiratory infectious diseases [23]. Together with a molecular virologic study, the surveillance data confirmed the emergence and expansion of the novel ON1 variant in 2020 during which the COVID-19–related public health measures were remained, even outcompeting influenza virus and adenovirus circulating in previous seasons, indeed caused an enormous increase in cases of medically attended respiratory infections by the end of 2020. However, the novel ON1 variant in 2020 did not show higher replication or virulence compared with those of a previously circulating genotype in our in vitro and in vivo models. These results need to be confirmed using more suitable model such as reverse genetics and primary cell in the future [24]. We hypothesized that RSV epidemic is related to antigenic variation of the 2020 novel ON1 variant, which is worth further investigating in the future.

Of note, an unprecedented low incidence of RSV infection during COVID-19 pandemic followed by out-of-season resurgence of RSV cases was observed worldwide such as Australia, Japan, and Israel [25,26,27]. The suppression of RSV activity in these countries coincided with strict public health measures and restrictions in response to increasing cases of COVID-19. The gradual relaxation of restrictions such as states border reopening, cessation of mandatory mask wearing, and school reopening contributed to the RSV resurgence in 2021 worldwide. Moreover, some experts stated that diminished immunity from a lack of exposure to RSV in the previous season may also lead to this consequence [27]. Although novel RSV-A lineages were dominant in the post-COVID-19 period in Australia, the scale of the RSV resurgence in Australia differed in states which were generally not greater than the usual winter season [26]. In contrast to the previous reports worldwide, the RSV outbreak in 2020 in Taiwan with 4-fold rise than those in 2010 occurred even under strict public measures and quarantine for overseas arrivals. Wu et al. demonstrated that one virus can induce interferon-stimulated immunity that block another viral infection [28]. The RSV outbreak in our study may because the number of children with respiratory viral infections has substantially decreased under non-pharmaceutical intervention during COVID-19 epidemic, contributing to the diminished interferon-stimulated immunity in children. Furthermore, a novel ON1 variant has emerged concurrently. Together with lack of interferon-stimulated antiviral immunity and the emergence of novel antigenicity, this variant may influence disease severity and viral transmission. Giving that spike in RSV cases is reported worldwide recently, tracking of evolving RSV variants and viral transmission is mandatory for verifying the effectiveness of vaccines or monoclonal antibodies.

## Contributors

YCH and KCT conceived the idea for this study. YCH, FTW, KCT, and WHL designed the study. WHL, FJL, WCL, WTT, ALT, and KTH prepared the data for analysis. WHL, FTW, CCK, YYC, HCL, YJP, analyzed and interpreted the data and prepared figures and tables. FTW, TUK, CHL, and CYL provide the data from Taiwan Centres for Disease Control. HCL and YYC, performed in vitro and in vivo assay and analysis. CWW performed pathological findings. WHL and YCH prepared the first draft of the manuscript. YCH had final responsibility for the decision to submit for publication. YCH, FTW, KCT provided suggestions on the design of the study and contributed to interpretation of results. All authors provided input to the overall direction and content of the paper, reviewed each draft of the paper, and reviewed and approved the final version.

## Supplementary Material

Supplemental Material

## Data Availability

The G protein and F protein sequences of RSV-A generated during this study were deposited into the GenBank database under accession number MZ417560−MZ417781 and OM801240−OM801242.

## Data sharing

De-identified patient datasets will be available upon written request to the corresponding author following publication.
