# Supplementary material for: Unprecedented outbreak of respiratory syncytial virus in Taiwan associated with ON1 variant emergence between 2010 and 2020
Source: Emerg Microbes Infect. 2022 Mar 31;11(1):1000–9. doi: 10.1080/22221751.2022.2054365 (PMC8979508; doi:10.1080/22221751.2022.2054365)
Supplement: Supplemental Material [file TEMI_A_2054365_SM3147.docx]

***Supplemental Appendix***

**Unprecedented outbreak of respiratory syncytial virus in Taiwan associated with ON1 variant emergence between 2010 and 2020**

Wei-Hsuan Lin, MD^1a^, Fang-Tzy Wu, PhD^2a^, Yi-Yin Chen, PhD^1^, Chih-Wei Wang^3^, MD, Ho-Chen Lin, PhD^1^, Ching-Chia Kuo, MD^1^, Wan-Chun Lai, MD^1^, Fang-Ju Lin, MD^4^, Wan-Tin Tiew, MD^1^, An-Li Tsai, MD^1^, Kuan-Ta Ho, MD^1^, Ting-Yu Kuo, MS^2^, Chung-Hao Li, MS^2^, Ching-Yi Wu, MS^2^, Yi-Jiun Pan, PhD^5^, Kuo-Chien Tsao, BS^6,7*^, Yu-Chia Hsieh, MD. PhD^1*^

*Corresponding author

Yu-Chia Hsieh

Department of Pediatrics, Linkou Chang Gung Memorial Hospital, No.5, Fuxing Street, Guishan District, Taoyuan City 333, Taiwan

(yuchiahsieh@gmail.com)

Kuo-Chien Tsao

Department of Laboratory Medicine, Linkou Chang Gung Memorial Hospital, No.5, Fuxing Street, Guishan District, Taoyuan City 333, Taiwan

(kctsao@cgmh.org.tw)

**Supplemental Methods**

***Surveillance network and viral isolation and identification***

Throat swabs were inoculated into human embryonic lung diploid fibroblast cells (MRC-5), human malignant embryonic rhabdomyosarcoma (RD) cells, and MDCK cells. Cultures were maintained in minimal essential media containing antibiotics and incubated at 35 °C. The inoculated cell cultures were maintained and observed for the presence of CPEs for at least 4 weeks. Respiratory viruses, including human adenovirus, human metapneumoniae virus, human parainfluenza virus type 1, 2, and 3, influenza A and B viruses, and RSV in CPE-positive cases were identified using immunofluorescence assay with D3 Ultra DFA respiratory virus screening and identification kit (Diagnostic Hybrids, Inc., Athens, OH, USA). The RSV-positive culture was further passaged for 3 times. Then the culture supernatants were collected, aliquoted in 0.1mL, and stored at -80°C.

***RSV isolation, typing, and sequencing***

Viral RNA from frozen stock was extracted using QIAamp Viral RNA Mini Kit (QIAGEN) and reverse-transcribed into cDNA by using the SuperScript™ III First-Strand Synthesis System (Invitrogen™) according to the manual instructions. The RSV G protein of viral cDNA was amplified by polymerase chain reaction (PCR) using Taq DNA Polymerase High Fidelity (Platinum™) with primers RSVA-GF, GGG CAA ATG CAA MCA TGT and RSVA-GR, GTT ATR ACA CTR GTA TAC CAA CC. The PCR amplicons were sequenced by Sanger method then differentiated into RSV types (type A and B) based on NCBI nucleotide database. The full-length gene sequence of the F protein of RSV-A was also sequenced as previously described [1]. Gene sequences of G and F proteins were assembled using Sequencher (V4.0.1) and the assembled contigs were deposited in GenBank database (accession numbers MZ417560− MZ417781 and OM801240− OM801242, listed in Tables S1 and S2). The phylogenetic tree was constructed by MEGA 6 using the maximum likelihood method based on the Haswgawa-Kishino-Yano model and a discrete Gamma distribution with 5 categories (+G) to model evolutionary rate differences among sites, with bootstrap values of 1000 (alignment reference sequences: JN257693.1 and JX198112.1 for G protein and F protein, respectively).

***Viral RNA assay***

RT-qPCR analysis was performed using the following primers and probe: RSV-3255F: 5’-GGG CAA ATA TGG AAA CAT AC-3’ and RSV-3370R: 5’-CAC CCA TAT TGT WAG TGA TGC-3’, RSV-3287P probe: 5’-FAM-CAC GAR GGC TCC ACA TAC ACA-QSY-3’ on the QuantStudio 7 real-time system (Applied Biosystems) following the manufacturer’s protocols.

***Pathological score***

For histopathological analysis, lung tissue was fixed in 10% buffered formalin and then embedded in paraffin. Slides with hematoxylin-eosin and Masson's Trichrome stain were prepared by the pathology core of Chang Gung Memorial Hospital, analyzed as previously described [2].

***Statistical analyses***

Mean values with standard deviations were calculated for continuous variables, and percentages were used for categorical variables. For continuous variables, comparisons of means were conducted using t-test and one-way independent analysis of variance (ANOVA) with post-hoc analysis. For categorical variables, the chi-squared test or Fisher’s exact test was used. The annual rates of RSV, adenovirus, and influenza virus positivity were compared using logistic regression. Univariate analysis followed by multivariate analysis (logistic regression) was used to assess the risk factors, including sex, age, wheezing, ON1 in 2020, white blood cell count (WBC), and C-reactive protein (CRP) value, with oxygen saturation <94% during hospitalization. Variables with p <0.1, in the univariate analysis, were included in the multivariate analysis. For animal body weight changes and histopathologic scoring, data sets involving more than two groups are assessed by one-way analysis of variance, followed by non-parametric Kruskal-Wallis test. Data sets involving two groups are assessed by non-parametric Mann-Whitney test.

**Table S1.** RVS-A G gene sequences used in this study.

| **Strain name** | **Year** | **Area** | **Accession number** | **Reference** |
| --- | --- | --- | --- | --- |
| ON67-1210A | 2010 | Canada | JN257693.1 | [3] |
| 1302-319AN | 2013 | Italy | KC858211.1 | Unpublished |
| KEN/Kilifi/1281_30/12/2014 | 2014 | Kenya | KX453458.1 | [4] |
| KEN/Kilifi/1255_08/12/2014 | 2014 | Kenya | KX453432.1 | [4] |
| R100_61_ON1-TWN-2011 | 2011 | Taiwan | MH045601 | [5] |
| R101-33-GA-TWN-2012 | 2012 | Taiwan | MF496488 | [5] |
| M103-103-GA-TWN-2013 | 2013 | Taiwan | MF496535 | [5] |
| M103-172-GA-TWN-2013 | 2013 | Taiwan | MF496377 | [5] |
| M103-249-GA-TWN-2013 | 2013 | Taiwan | MF496378 | [5] |
| M103-323-GA-TWN-2013 | 2013 | Taiwan | MF496379 | [5] |
| M103-415-GA-TWN-2013 | 2013 | Taiwan | MF496380 | [5] |
| M103-458-GA-TWN-2013 | 2013 | Taiwan | MF496381 | [5] |
| M103-471-GA-TWN-2013 | 2013 | Taiwan | MF496382 | [5] |
| M103-599-GA-TWN-2013 | 2013 | Taiwan | MF496383 | [5] |
| M103-655-GA-TWN-2013 | 2013 | Taiwan | MF496384 | [5] |
| M103-670-GA-TWN-2013 | 2013 | Taiwan | MF496385 | [5] |
| M103-83-GA-TWN-2013 | 2013 | Taiwan | MF496521 | [5] |
| PC-52-GA-TWN-2013 | 2013 | Taiwan | MF496386 | [5] |
| PC-55-GA-TWN-2013 | 2013 | Taiwan | MF496387 | [5] |
| PC-66-GA-TWN-2013 | 2013 | Taiwan | MF496388 | [5] |
| R102-06-GA-TWN-2013 | 2013 | Taiwan | MF496486 | [5] |
| R102-17-GA-TWN-2013 | 2013 | Taiwan | MF496396 | [5] |
| R102-18-GA-TWN-2013 | 2013 | Taiwan | MF496397 | [5] |
| R102-21-GA-TWN-2013 | 2013 | Taiwan | MF496398 | [5] |
| M103-16-GA-TWN-2014 | 2014 | Taiwan | MF496529 | [5] |
| M103-17-GA-TWN-2014 | 2014 | Taiwan | MF496528 | [5] |
| M103-121-GA-TWN-2014 | 2014 | Taiwan | MF496534 | [5] |
| M103-132-GA-TWN-2014 | 2014 | Taiwan | MF496533 | [5] |
| M103-133-GA-TWN-2014 | 2014 | Taiwan | MF496532 | [5] |
| M103-135-GA-TWN-2014 | 2014 | Taiwan | MF496531 | [5] |
| M103-144-GA-TWN-2014 | 2014 | Taiwan | MF496530 | [5] |
| M103-274-GA-TWN-2014 | 2014 | Taiwan | MF496527 | [5] |
| M103-279-GA-TWN-2014 | 2014 | Taiwan | MF496526 | [5] |
| M103-487-GA-TWN-2015 | 2015 | Taiwan | MF496525 | [5] |
| M103-582-GA-TWN-2015 | 2015 | Taiwan | MF496524 | [5] |
| M103-633-GA-TWN-2015 | 2015 | Taiwan | MF496523 | [5] |
| M103-656-GA-TWN-2015 | 2015 | Taiwan | MF496522 | [5] |
| R104-04-GA-TWN-2015 | 2015 | Taiwan | MF496485 | [5] |
| R104-05-GA-TWN-2015 | 2015 | Taiwan | MF496484 | [5] |
| R104-09-GA-TWN-2015 | 2015 | Taiwan | MF496483 | [5] |
| R104-13-GA-TWN-2015 | 2015 | Taiwan | MF496482 | [5] |
| R104-14-GA-TWN-2015 | 2015 | Taiwan | MF496481 | [5] |
| R104-17-GA-TWN-2015 | 2015 | Taiwan | MF496480 | [5] |
| R104-19-GA-TWN-2015 | 2015 | Taiwan | MF496479 | [5] |
| R104-20-GA-TWN-2015 | 2015 | Taiwan | MF496478 | [5] |
| R104-25-GA-TWN-2015 | 2015 | Taiwan | MF496477 | [5] |
| R104-27-GA-TWN-2015 | 2015 | Taiwan | MF496476 | [5] |
| R104-29-GA-TWN-2015 | 2015 | Taiwan | MF496475 | [5] |
| R105-01-GA-TWN-2016 | 2016 | Taiwan | MF496474 | [5] |
| R105-03-GA-TWN-2016 | 2016 | Taiwan | MF496473 | [5] |
| TW/LH/00973/2018 | 2018 | Taiwan | MZ417560 | this study |
| TW/LH/00981/2018 | 2018 | Taiwan | MZ417561 | this study |
| TW/LH/01023/2018 | 2018 | Taiwan | MZ417562 | this study |
| TW/LH/01245/2018 | 2018 | Taiwan | MZ417563 | this study |
| TW/LH/01367/2018 | 2018 | Taiwan | MZ417564 | this study |
| TW/LH/01442/2018 | 2018 | Taiwan | MZ417565 | this study |
| TW/LH/01530/2018 | 2018 | Taiwan | MZ417566 | this study |
| TW/LH/01669/2018 | 2018 | Taiwan | MZ417567 | this study |
| TW/LH/01769/2018 | 2018 | Taiwan | MZ417568 | this study |
| TW/LH/50517/2018 | 2018 | Taiwan | MZ417569 | this study |
| TW/LH/01977/2018 | 2018 | Taiwan | MZ417570 | this study |
| TW/LH/02114/2018 | 2018 | Taiwan | MZ417571 | this study |
| TW/LH/50668/2018 | 2018 | Taiwan | MZ417572 | this study |
| TW/LH/02147/2018 | 2018 | Taiwan | MZ417573 | this study |
| TW/LH/02242/2018 | 2018 | Taiwan | MZ417574 | this study |
| TW/LH/02412/2018 | 2018 | Taiwan | MZ417575 | this study |
| TW/LH/50810/2018 | 2018 | Taiwan | MZ417576 | this study |
| TW/LH/02632/2018 | 2018 | Taiwan | MZ417577 | this study |
| TW/LH/02705/2018 | 2018 | Taiwan | MZ417578 | this study |
| TW/LH/02731/2018 | 2018 | Taiwan | MZ417579 | this study |
| TW/LH/02904/2018 | 2018 | Taiwan | MZ417580 | this study |
| TW/LH/02926/2018 | 2018 | Taiwan | MZ417581 | this study |
| TW/LH/02978/2018 | 2018 | Taiwan | MZ417582 | this study |
| TW/KH/40009/2018 | 2018 | Taiwan | MZ417583 | this study |
| TW/LH/03023/2018 | 2018 | Taiwan | MZ417584 | this study |
| TW/LH/03117/2018 | 2018 | Taiwan | MZ417585 | this study |
| TW/LH/03164/2018 | 2018 | Taiwan | MZ417586 | this study |
| TW/LH/03291/2018 | 2018 | Taiwan | MZ417587 | this study |
| TW/LH/740006/2018 | 2018 | Taiwan | MZ417588 | this study |
| TW/LH/03498/2018 | 2018 | Taiwan | MZ417589 | this study |
| TW/LH/51007/2018 | 2018 | Taiwan | MZ417590 | this study |
| TW/LH/03524/2018 | 2018 | Taiwan | MZ417591 | this study |
| TW/KH/140005/2018 | 2018 | Taiwan | MZ417592 | this study |
| TW/LH/03545/2018 | 2018 | Taiwan | MZ417593 | this study |
| TW/LH/51031/2018 | 2018 | Taiwan | MZ417594 | this study |
| TW/LH/03639/2018 | 2018 | Taiwan | MZ417595 | this study |
| TW/LH/40005/2018 | 2018 | Taiwan | MZ417596 | this study |
| TW/LH/103667/2018 | 2018 | Taiwan | MZ417597 | this study |
| TW/LH/03761/2018 | 2018 | Taiwan | MZ417598 | this study |
| TW/LH/03827/2018 | 2018 | Taiwan | MZ417599 | this study |
| TW/LH/03910/2018 | 2018 | Taiwan | MZ417600 | this study |
| TW/LH/03983/2018 | 2018 | Taiwan | MZ417601 | this study |
| TW/LH/04273/2018 | 2018 | Taiwan | MZ417602 | this study |
| TW/LH/04309/2018 | 2018 | Taiwan | MZ417603 | this study |
| TW/LH/04112/2018 | 2018 | Taiwan | MZ417604 | this study |
| TW/LH/51179/2018 | 2018 | Taiwan | MZ417605 | this study |
| TW/LH/04600/2018 | 2018 | Taiwan | MZ417606 | this study |
| TW/LH/00719/2018 | 2018 | Taiwan | MZ417607 | this study |
| TW/LH/01113/2018 | 2018 | Taiwan | MZ417608 | this study |
| TW/LH/01344/2019 | 2019 | Taiwan | MZ417609 | this study |
| TW/LH/01349/2019 | 2019 | Taiwan | MZ417610 | this study |
| TW/LH/01495/2019 | 2019 | Taiwan | MZ417611 | this study |
| TW/LH/0646/2019 | 2019 | Taiwan | MZ417612 | this study |
| TW/LH/01688/2019 | 2019 | Taiwan | MZ417613 | this study |
| TW/LH/50431/2019 | 2019 | Taiwan | MZ417614 | this study |
| TW/LH/01798/2019 | 2019 | Taiwan | MZ417615 | this study |
| TW/KH/40013/2019 | 2019 | Taiwan | MZ417616 | this study |
| TW/LH/02748/2019 | 2019 | Taiwan | MZ417617 | this study |
| TW/LH/02786/2019 | 2019 | Taiwan | MZ417618 | this study |
| TW/KH/40020/2019 | 2019 | Taiwan | MZ417619 | this study |
| TW/KH/340005/2019 | 2019 | Taiwan | MZ417620 | this study |
| TW/KH/840008/2019 | 2019 | Taiwan | MZ417621 | this study |
| TW/KH/940008/2019 | 2019 | Taiwan | MZ417622 | this study |
| TW/LH/03085/2019 | 2019 | Taiwan | MZ417623 | this study |
| TW/KH/40012/2019 | 2019 | Taiwan | MZ417624 | this study |
| TW/LH/03097/2019 | 2019 | Taiwan | MZ417625 | this study |
| TW/LH/03156/2019 | 2019 | Taiwan | MZ417626 | this study |
| TW/KH/40014/2019 | 2019 | Taiwan | MZ417627 | this study |
| TW/LH/03215/2019 | 2019 | Taiwan | MZ417628 | this study |
| TW/LH/03234/2019 | 2019 | Taiwan | MZ417629 | this study |
| TW/LH/03242/2019 | 2019 | Taiwan | MZ417630 | this study |
| TW/LH/03340/2019 | 2019 | Taiwan | MZ417631 | this study |
| TW/LH/03442/2019 | 2019 | Taiwan | MZ417632 | this study |
| TW/LH/03487/2019 | 2019 | Taiwan | MZ417633 | this study |
| TW/LH/03491/2019 | 2019 | Taiwan | MZ417634 | this study |
| TW/LH/03592/2019 | 2019 | Taiwan | MZ417635 | this study |
| TW/LH/03679/2019 | 2019 | Taiwan | MZ417636 | this study |
| TW/LH/03746/2019 | 2019 | Taiwan | MZ417637 | this study |
| TW/LH/03751/2019 | 2019 | Taiwan | MZ417638 | this study |
| TW/LH/40003/2019 | 2019 | Taiwan | MZ417639 | this study |
| TW/LH/03765/2019 | 2019 | Taiwan | MZ417640 | this study |
| TW/LH/03786/2019 | 2019 | Taiwan | MZ417641 | this study |
| TW/KH/40025/2019 | 2019 | Taiwan | MZ417642 | this study |
| TW/LH/03849/2019 | 2019 | Taiwan | MZ417643 | this study |
| TW/LH/03946/2019 | 2019 | Taiwan | MZ417644 | this study |
| TW/LH/03952/2019 | 2019 | Taiwan | MZ417645 | this study |
| TW/LH/04046/2019 | 2019 | Taiwan | MZ417646 | this study |
| TW/LH/04085/2019 | 2019 | Taiwan | MZ417647 | this study |
| TW/LH/04176/2019 | 2019 | Taiwan | MZ417648 | this study |
| TW/LH/04207/2019 | 2019 | Taiwan | MZ417649 | this study |
| TW/LH/04229/2019 | 2019 | Taiwan | MZ417650 | this study |
| TW/LH/04316/2019 | 2019 | Taiwan | MZ417651 | this study |
| TW/LH/04554/2019 | 2019 | Taiwan | MZ417652 | this study |
| TW/LH/04692/2019 | 2019 | Taiwan | MZ417653 | this study |
| TW/LH/04698/2019 | 2019 | Taiwan | MZ417654 | this study |
| TW/LH/05019/2019 | 2019 | Taiwan | MZ417655 | this study |
| 2019-RSV-00038 | 2019 | Taiwan | OM801242 | this study |
| TW/LH/50058/2020 | 2020 | Taiwan | MZ417656 | this study |
| TW/LH/50259/2020 | 2020 | Taiwan | MZ417657 | this study |
| TW/LH/50281/2020 | 2020 | Taiwan | MZ417658 | this study |
| TW/LH/02756/2020 | 2020 | Taiwan | MZ417659 | this study |
| TW/LH/02761/2020 | 2020 | Taiwan | MZ417660 | this study |
| TW/LH/02827/2020 | 2020 | Taiwan | MZ417661 | this study |
| TW/LH/02844/2020 | 2020 | Taiwan | MZ417662 | this study |
| TW/LH/02868/2020 | 2020 | Taiwan | MZ417663 | this study |
| TW/LH/50333/2020 | 2020 | Taiwan | MZ417664 | this study |
| TW/LH/50337/2020 | 2020 | Taiwan | MZ417665 | this study |
| TW/LH/03020/2020 | 2020 | Taiwan | MZ417666 | this study |
| TW/LH/50356/2020 | 2020 | Taiwan | MZ417667 | this study |
| TW/LH/03052/2020 | 2020 | Taiwan | MZ417668 | this study |
| TW/LH/03055/2020 | 2020 | Taiwan | MZ417669 | this study |
| TW/LH/03061/2020 | 2020 | Taiwan | MZ417670 | this study |
| TW/LH/03062/2020 | 2020 | Taiwan | MZ417671 | this study |
| TW/LH/03114/2020 | 2020 | Taiwan | MZ417672 | this study |
| TW/LH/50381/2020 | 2020 | Taiwan | MZ417673 | this study |
| TW/LH/50395/2020 | 2020 | Taiwan | MZ417674 | this study |
| TW/LH/03253/2020 | 2020 | Taiwan | MZ417675 | this study |
| TW/LH/03254/2020 | 2020 | Taiwan | MZ417676 | this study |
| TW/LH/03266/2020 | 2020 | Taiwan | MZ417677 | this study |
| TW/LH/03282/2020 | 2020 | Taiwan | MZ417678 | this study |
| TW/LH/03284/2020 | 2020 | Taiwan | MZ417679 | this study |
| TW/LH/03287/2020 | 2020 | Taiwan | MZ417680 | this study |
| TW/LH/03298/2020 | 2020 | Taiwan | MZ417681 | this study |
| TW/LH/03306/2020 | 2020 | Taiwan | MZ417682 | this study |
| TW/LH/03314/2020 | 2020 | Taiwan | MZ417683 | this study |
| TW/LH/03315/2020 | 2020 | Taiwan | MZ417684 | this study |
| TW/LH/03316/2020 | 2020 | Taiwan | MZ417685 | this study |
| TW/LH/03330/2020 | 2020 | Taiwan | MZ417686 | this study |
| TW/LH/50432/2020 | 2020 | Taiwan | MZ417687 | this study |
| TW/LH/03338/2020 | 2020 | Taiwan | MZ417688 | this study |
| TW/LH/03358/2020 | 2020 | Taiwan | MZ417689 | this study |
| TW/LH/50445/2020 | 2020 | Taiwan | MZ417690 | this study |
| TW/LH/03368/2020 | 2020 | Taiwan | MZ417691 | this study |
| TW/LH/03383/2020 | 2020 | Taiwan | MZ417692 | this study |
| TW/LH/03387/2020 | 2020 | Taiwan | MZ417693 | this study |
| TW/LH/03388/2020 | 2020 | Taiwan | MZ417694 | this study |
| TW/LH/03395/2020 | 2020 | Taiwan | MZ417695 | this study |
| TW/LH/03398/2020 | 2020 | Taiwan | MZ417696 | this study |
| TW/LH/03402/2020 | 2020 | Taiwan | MZ417697 | this study |
| TW/LH/03420/2020 | 2020 | Taiwan | MZ417698 | this study |
| TW/LH/03436/2020 | 2020 | Taiwan | MZ417699 | this study |
| TW/LH/03448/2020 | 2020 | Taiwan | MZ417700 | this study |
| TW/LH/03451/2020 | 2020 | Taiwan | MZ417701 | this study |
| TW/LH/03453/2020 | 2020 | Taiwan | MZ417702 | this study |
| TW/LH/50465/2020 | 2020 | Taiwan | MZ417703 | this study |
| TW/LH/03482/2020 | 2020 | Taiwan | MZ417704 | this study |
| TW/LH/03483/2020 | 2020 | Taiwan | MZ417705 | this study |
| TW/LH/50466/2020 | 2020 | Taiwan | MZ417706 | this study |
| TW/LH/03492/2020 | 2020 | Taiwan | MZ417707 | this study |
| TW/LH/50475/2020 | 2020 | Taiwan | MZ417708 | this study |
| TW/LH/03503/2020 | 2020 | Taiwan | MZ417709 | this study |
| TW/LH/03525/2020 | 2020 | Taiwan | MZ417710 | this study |
| TW/LH/50492/2020 | 2020 | Taiwan | MZ417711 | this study |
| TW/LH/50493/2020 | 2020 | Taiwan | MZ417712 | this study |
| TW/LH/50494/2020 | 2020 | Taiwan | MZ417713 | this study |
| TW/LH/50508/2020 | 2020 | Taiwan | MZ417714 | this study |
| TW/LH/50510/2020 | 2020 | Taiwan | MZ417715 | this study |
| TW/LH/03579/2020 | 2020 | Taiwan | MZ417716 | this study |
| TW/LH/03588/2020 | 2020 | Taiwan | MZ417717 | this study |
| TW/LH/50524/2020 | 2020 | Taiwan | MZ417718 | this study |
| TW/LH/03607/2020 | 2020 | Taiwan | MZ417719 | this study |
| TW/LH/03620/2020 | 2020 | Taiwan | MZ417720 | this study |
| TW/LH/50533/2020 | 2020 | Taiwan | MZ417721 | this study |
| TW/LH/50536/2020 | 2020 | Taiwan | MZ417722 | this study |
| TW/LH/03636/2020 | 2020 | Taiwan | MZ417723 | this study |
| TW/LH/03652/2020 | 2020 | Taiwan | MZ417724 | this study |
| TW/LH/403667/2020 | 2020 | Taiwan | MZ417725 | this study |
| TW/LH/50542/2020 | 2020 | Taiwan | MZ417726 | this study |
| TW/LH/50543/2020 | 2020 | Taiwan | MZ417727 | this study |
| TW/LH/03700/2020 | 2020 | Taiwan | MZ417728 | this study |
| TW/LH/03715/2020 | 2020 | Taiwan | MZ417729 | this study |
| TW/LH/50554/2020 | 2020 | Taiwan | MZ417730 | this study |
| TW/LH/03736/2020 | 2020 | Taiwan | MZ417731 | this study |
| TW/LH/03759/2020 | 2020 | Taiwan | MZ417732 | this study |
| TW/LH/03744/2020 | 2020 | Taiwan | MZ417733 | this study |
| TW/LH/03805/2020 | 2020 | Taiwan | MZ417734 | this study |
| TW/LH/03870/2020 | 2020 | Taiwan | MZ417735 | this study |
| TW/LH/03906/2020 | 2020 | Taiwan | MZ417736 | this study |
| TW/KH/00097/2020 | 2020 | Taiwan | MZ417737 | this study |
| TW/KH/02516/2020 | 2020 | Taiwan | MZ417738 | this study |
| TW/KH/02538/2020 | 2020 | Taiwan | MZ417739 | this study |
| TW/KH/02541/2020 | 2020 | Taiwan | MZ417740 | this study |
| TW/KH/02554/2020 | 2020 | Taiwan | MZ417741 | this study |
| TW/KH/02614/2020 | 2020 | Taiwan | MZ417742 | this study |
| TW/KH/02694/2020 | 2020 | Taiwan | MZ417743 | this study |
| TW/KH/02714/2020 | 2020 | Taiwan | MZ417744 | this study |
| TW/KH/02376/2020 | 2020 | Taiwan | MZ417745 | this study |
| TW/KH/02442/2020 | 2020 | Taiwan | MZ417746 | this study |
| 2020-RSV-E115 | 2020 | Taiwan | OM801240 | this study |
| 2020-RSV-E001 | 2021 | Taiwan | OM801241 | this study |
| TW/LH/00059/2021 | 2021 | Taiwan | MZ417747 | this study |
| TW/LH/00063/2021 | 2021 | Taiwan | MZ417748 | this study |
| TW/LH/00068/2021 | 2021 | Taiwan | MZ417749 | this study |
| TW/LH/00116/2021 | 2021 | Taiwan | MZ417750 | this study |
| TW/LH/00133/2021 | 2021 | Taiwan | MZ417751 | this study |
| TW/LH/00170/2021 | 2021 | Taiwan | MZ417752 | this study |

**Table S2.** RVS-A F gene sequences used in this study.

| **Strain name** | **Year** | **Area** | **Accession number** | **Reference** |
| --- | --- | --- | --- | --- |
| AGG39392.1 | 1956 | USA | JX198112.1 | [6] |
| TW/LH/01179/2018 | 2018 | Taiwan | MZ417753 | this study |
| TW/LH/0977/2018 | 2018 | Taiwan | MZ417754 | this study |
| TW/LH/02412/2018 | 2018 | Taiwan | MZ417755 | this study |
| TW/LH/03164/2018 | 2018 | Taiwan | MZ417756 | this study |
| TW/LH/03910/2018 | 2018 | Taiwan | MZ417757 | this study |
| TW/LH/04273/2018 | 2018 | Taiwan | MZ417758 | this study |
| TW/LH/04309/2018 | 2018 | Taiwan | MZ417759 | this study |
| TW/LH/04412/2018 | 2018 | Taiwan | MZ417760 | this study |
| TW/LH/01113/2019 | 2019 | Taiwan | MZ417761 | this study |
| TW/LH/01349/2019 | 2019 | Taiwan | MZ417762 | this study |
| TW/LH/0798/2019 | 2019 | Taiwan | MZ417763 | this study |
| TW/LH/02786/2019 | 2019 | Taiwan | MZ417764 | this study |
| TW/LH/03097/2019 | 2019 | Taiwan | MZ417765 | this study |
| TW/LH/03234/2019 | 2019 | Taiwan | MZ417766 | this study |
| TW/LH/03442/2019 | 2019 | Taiwan | MZ417767 | this study |
| TW/LH/03769/2019 | 2019 | Taiwan | MZ417768 | this study |
| TW/LH/04085/2019 | 2019 | Taiwan | MZ417769 | this study |
| TW/LH/04692/2019 | 2019 | Taiwan | MZ417770 | this study |
| TW/LH/02844/2020 | 2020 | Taiwan | MZ417771 | this study |
| TW/LH/03020/2020 | 2020 | Taiwan | MZ417772 | this study |
| TW/LH/03253/2020 | 2020 | Taiwan | MZ417773 | this study |
| TW/LH/03451/2020 | 2020 | Taiwan | MZ417774 | this study |
| TW/LH/03607/2020 | 2020 | Taiwan | MZ417775 | this study |
| TW/LH/04600/2020 | 2020 | Taiwan | MZ417776 | this study |
| TW/LH/03906/2020 | 2020 | Taiwan | MZ417777 | this study |
| TW/LH/50259/2020 | 2020 | Taiwan | MZ417778 | this study |
| TW/LH/50333/2020 | 2020 | Taiwan | MZ417779 | this study |
| TW/LH/50432/2020 | 2020 | Taiwan | MZ417780 | this study |
| TW/LH/50542/2020 | 2020 | Taiwan | MZ417781 | this study |

**Table S3.** Between and Within Group Average Distance Estimation

| Epidemic Seasons | Number of Group | p Distance Within Group | Epidemic Seasons | p Distance Between Group |
| --- | --- | --- | --- | --- |
| 2018 | 47 | 0.023 | 2018 vs 2019 | 0.029 |
| 2019 | 49 | 0.023 | 2019 vs 2020 | 0.021 |
| 2020 | 97 | 0.005 |  |  |

The average P distance among nucleotide sequences of the 3 epidemic seasons was calculated by pairwise comparison using Tamura-Nei model, gamma distributed including transitions and transversions, in MEGA 6.

**Table S4**. Histopathologic scores of lung tissue after RSV-A infection ^a^

| Strains | Groups  (DPI) | Epithelia damage  (E) | P value | Interstitial cellularity  (I) | P value | Peribronchovascular infiltrates  (P) | P value |
| --- | --- | --- | --- | --- | --- | --- | --- |
| Healthy control | Day 6 | 1.5 ± 1.0 |  | 1.0 ± 0 |  | 0 ± 0 |  |
| 2018/RSV-A  (51031) | Day 2 | 1.5 ± 1 |  | 1.25 ± 0.5 |  | 0 ± 0 |  |
|  | Day 4 | 2 ± 0.8 |  | 1.25 ± 0.5 |  | 0 ± 0 |  |
|  | Day 6 | 1.75 ± 1.3 |  | 1.0 ± 0 |  | 0 ± 0 |  |
| 2020/RSV-A  (E115) | Day 2 | 2.5 ± 0.6 | 0.1 | 1.0 ± 0 | 0.3 | 0 ± 0 | 1.0 |
|  | Day 4 | 2.75 ± 0.5 | 0.2 | 1.0 ± 0 | 0.3 | 0 ± 0 | 1.0 |
|  | Day 6 | 2.75 ± 0.5 | 0.1 | 1.0 ± 0 | 1.0 | 0 ± 0 | 1.0 |

^a^ Each value represents the mean ± SD of 6 fields of 2 individual lung sections from each mouse in a group (n=4 for each group).

* P value was compared to 2018/RSV-A at Day 2 and Day 4; to 2018/RSV-A and healthy control at Day 6.

The average value for two groups is shown as no statistically significant difference was observed.

DPI: day post virus infection.


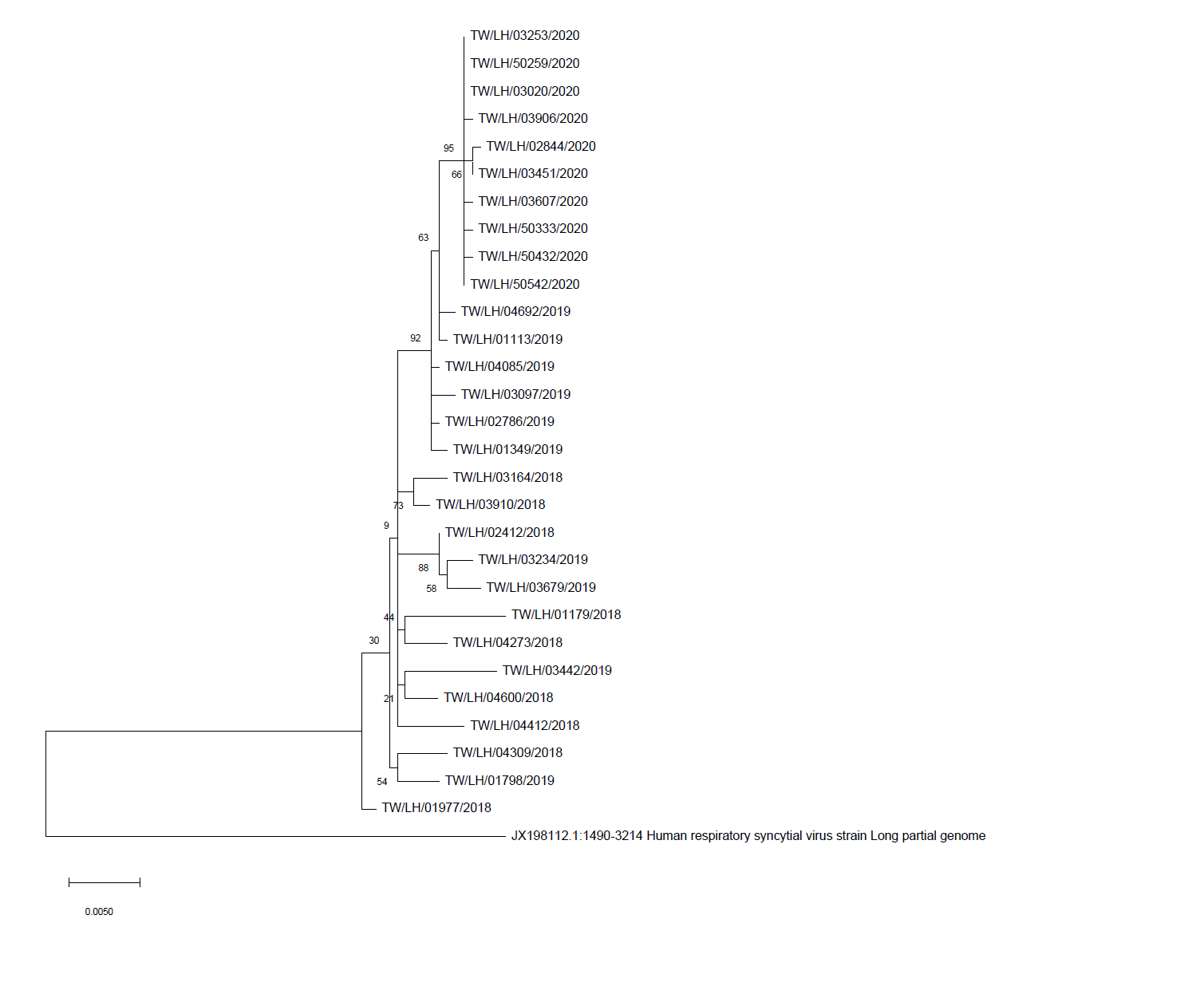


**Figure S1.** The phylogenetic tree based on F gene sequences of RSV-A from 2018 to 2020. Most strains of the 2020 season clustered apart from those of the previous seasons and reference strain (JX198112.1). Scale bar shows the number of substitutions per site.


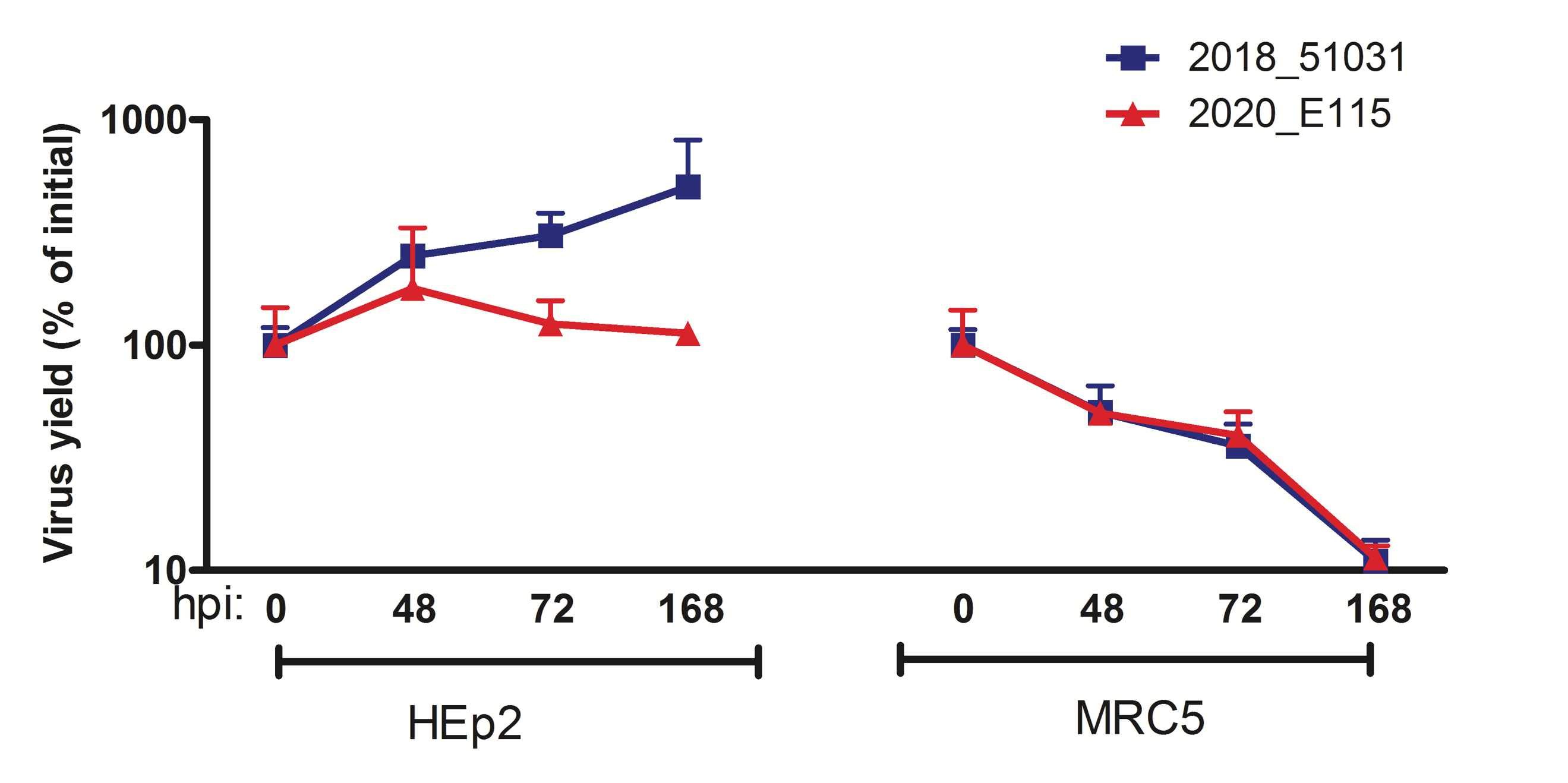
**Figure S2**. Viral replication of 2018/RSV-A isolates (51031) and 2021/RSV-A isolates (E115) in different cell types. Viral replication is presented as the percentage of initial RNA copies of 2018/RSVA (blue line) and 2020/RSVA (red line) viruses produced from different cell types. Cells were infected with RSV-A isolates at a 2×10^7^ RNA copies. Virus yield in the culture medium were determined by quantitative RT-PCR (RT-qPCR). Data are shown as the mean ± standard deviation (SD), n=3 in each group. Hpi: hours post infection.

**Figure S3.** BALB/c mice were intranasally infected with 1×10^7^ RNA copies /50 μL of 2018/RSV-A or 2020/RSV-A. (A) Weight changes over six days after virus infection, n=4 in each group. No significantly change between mock and virus infection groups (p> 0.05). (B) Amount of virus yield as measured from RT-qPCR results of the murine nasal wash on day 2,4, and 6 after infection. Data are shown as the mean ± standard deviation (SD), n=4 in each group. (C) Amount of virus yield per gram of lung tissue as measured from RT-qPCR results of the murine whole lung homogeneous on day 2,4, and 6 after infection. Data are shown as the mean ± standard deviation (SD), n=4 in each group. No significantly difference between 2018/RSV-A and 2020/RSV-A in nasal wash and lung after infection (p> 0.05).

**Figure S4.** Lung tissue section of healthy control (A−C), 2018/RSV-A (D−F), and 2020/RSV-A (G−I) infected mice on day 6 post infection. Arrow indicates epithelial damage of panel A, D, and G. Arrow indicates interstitial cellularity of panel B, E, and H. Arrow indicates peribronchovascular infiltrates of panel C, F, and I (hematoxylin and eosin stain, 400×).

**Figure S5.** Lung tissue section of healthy control, 2018/RSV-A, and 2020/RSV-A infected mice on day 6 post infection (Masson's Trichrome stain, 400×). No fibrosis is seen in all three groups.

**Supplementary Reference:**

1. Bin L, Liu H, Tabor DE, et al. Emergence of new antigenic epitopes in the glycoproteins of human respiratory syncytial virus collected from a US surveillance study, 2015–17. Scientific Reports. 2019 2019/03/07;9(1):3898.

2. Kumar M, Behera AK, Matsuse H, et al. Intranasal IFN-gamma gene transfer protects BALB/c mice against respiratory syncytial virus infection. Vaccine. 1999 Oct 14;18(5-6):558-67.

3. Eshaghi A, Duvvuri VR, Lai R, et al. Genetic variability of human respiratory syncytial virus A strains circulating in Ontario: a novel genotype with a 72 nucleotide G gene duplication. PLoS One. 2012;7(3):e32807.

4. Otieno JR, Kamau EM, Agoti CN, et al. Spread and Evolution of Respiratory Syncytial Virus A Genotype ON1, Coastal Kenya, 2010-2015. Emerg Infect Dis. 2017 Feb;23(2):264-271.

5. Chi H, Hsiao KL, Weng LC, et al. Persistence and continuous evolution of the human respiratory syncytial virus in northern Taiwan for two decades. Sci Rep. 2019 Mar 18;9(1):4704.

6. Tapia LI, Shaw CA, Aideyan LO, et al. Gene sequence variability of the three surface proteins of human respiratory syncytial virus (HRSV) in Texas. PLoS One. 2014;9(3):e90786.
